# Cytometric measurement of *in vitro* inhibition of *Plasmodium falciparum* field isolates by drugs: a new approach for re-invasion inhibition study

**DOI:** 10.1186/1475-2875-13-110

**Published:** 2014-03-21

**Authors:** Marie Louise Varela, Romy Razakandrainibe, Delphine Aldebert, Jean Christophe Barale, Ronan Jambou

**Affiliations:** 1 Institut Pasteur de Dakar, P O Box 220, Dakar, Senegal; 2 Unité d’immunologie Institut Pasteur de Madagascar, Antananarivo BP1274, Madagascar; 3 Laboratoire Adaptation et Pathogénie des Microorganismes, UJF-CNRS, Paris 5163, France; 4 Unité d'Immunologie Moléculaire des Parasites, Département de Parasitologie et de Mycologie, Institut Pasteur, CNRS URA, Paris 2581, France; 5 Present address: Institut Pasteur, Unité de Biologie et Génétique du Paludisme, Team “Malaria Targets and Drug Development”, Département de Parasitologie et de Mycologie, CNRS URA, 2581, F-75015 Paris, France; 6 Reseau International des Instituts Pasteur, Institut Pasteur, Paris, France

**Keywords:** *Plasmodium falciparum*, Flow cytometry, Drug sensitivity, Hydroethidine, Thiazole orange, Maturation, Re-invasion

## Abstract

**Background:**

A flow cytometric method is proposed to study *in vitro* drug sensitivity of *Plasmodium falciparum*. Standard [^3^H]-hypoxanthine incorporation assay gives only information on inhibition of maturation by drugs. This method is usable on field isolates and provides data on both inhibition of maturation and re-invasion.

**Methods:**

The method is based on the staining of parasites with hydroethidine (HE) and thiazole orange (TO) which allow differential identification of early, trophozoite and late stage of the parasite by flow cytometry. Late stages of the parasites are obtained by incubation in culture for 24 hours. Reinvasion is followed by culturing parasitized red blood cells for 24 h more.

**Results:**

Compared to the standard [^3^H]-hypoxanthine incorporation assay, it gave similar results as expressed by 50% inhibitory concentrations for chloroquine of laboratory strains and “field” isolates. The effect of quinine on the schizont-ring transition was also explored using this method. First data on the inhibition of re-invasion induced by quinine are presented for both *P. falciparum*-cultured strains and field isolates.

**Discussion:**

This method is simple to use event for field isolate study. It is suitable to analyse effect of drugs on steps of the parasite life cycle different for the maturation one. Using this method quinine was found to have a inhibitory effect on re-invasion of red cells by *Plasmodium*.

## Background

*Plasmodium falciparum* resistance to chloroquine and amodiaquine correlated with high morbidity and mortality. More recently *in vivo* resistance to artemisinin derivatives strengthens the need for malaria control programmes. To fight the burden associated with growing resistance to available anti-malarial drugs, the development of new drugs is mandatory, as well as surveying sensitivity to available drugs in field isolates [1]. Following theoretical mechanisms of action of drugs on *P. falciparum*, most *in vitro* drug sensitivity tests measure the effects on parasite growth [2]. Such tests allow the discovery of anti-malarial molecules, such as quinolines and artemisinin. Although new compounds that are particularly active on merozoite release and invasion steps are currently in development [3,4], very few tests address the impact of putative anti-malarial molecules on the schizont-merozoite transition or merozoite re-invasion step [5].

Besides the standard WHO macro test using count of schizonts by microscopy [6], the [^3^H] hypoxanthine microtest is the most widely used to assess *in vitro* anti-malarial properties of drugs [2]. This test quantifies the incorporation of radio-labelled compounds during DNA division. Immuno-enzymatic tests measuring release of HRP2 or LDH enzymes by the parasite are also available using final ELISA quantification of the enzymes in culture supernatant [7]. Microscopic examination of parasites is quite simple, but time consuming, with reliability depending on the technical skills of the operator, whereas, radio-labelling methods require expensive equipment and the use of radio isotopes difficult to manage in endemic areas. In addition, radio-labelling methods performed on patients’ blood need a careful removal of leukocytes to distinguish host cells from parasites growth, and are of no use for analysis of the different stages of the parasite’s life cycle. To counteract these difficulties, several dyes have been used to measure division of the nucleus by fluorimetry [8] or by flow cytometry. The most often used are: Hoechst 33258 [9], acridine orange [8,10,11], thiazole orange [12], hydroethidine [13], and recently YOYO-1 [5,14-16]. Sybergreen I based test was also standardized and is currently used in several laboratories [7]. All these tests quantify DNA to measure division in the parasite taking advantage of the absence of nucleus in human red blood cells. These techniques are used to count parasites in culture and *in vivo*[15]. To analyse the re-invasion step of the parasite’s life cycle, a method should provide information on the schizont-ring transition. The method proposed uses fluorescence dual labelling of the parasites using hydroethidine and thiazole. The use of this dual technique takes advantage of the labelling of both DNA and ARN by thiazole, whereas HE only labels doubled stranded DNA. Using DNA dye schizonte can easily be distinguished from other stages as the quantity of DNA is increasing, but trophozoite cannot thus be distinguished from early stage. HE/thiazole labelling allows this discrimination. This method is thus a simple and cheap cytometric approach to discriminate the different stages of the parasite’s life cycle and study both the inhibition of maturation of the parasite and the inhibition of re-invasion [17].

The protocol was standardized using diisopropylfluorophosphate (DFP) as positive control for inhibition of re-invasion and quinine was tested as a reinvasion inhibitor. DFP is a potent inhibitor of serine proteases, such as trypsin and chymotrypsin, which can inhibit merozoite release and re-invasion [18], and were found to inhibit uptake of haemoglobin by the parasite [19], choline influx [20] and haem polymerization. However, besides this activity, it is a well-known inhibitor of Ca2 + −activated K + Gardos channel [21-24] and of the K-Cl cotransport [25,26] in human erythrocytes. It also has an effect on the infected erythrocyte membrane [27] and its ability to inhibit *Plasmodium knowlesi* invasion was also described [28]. These activities are not under the control of the parasite (see [29] for review), which could explain the very low rate of quinine resistance reported worldwide, despite 300 years of use.

## Methods

### IRBC cell culture

*Plasmodium falciparum* 3D7 (chloroquine sensitive), Palo Alto (chloroquine sensitive), and FCM 29 (chloroquine-resistant strain from Cameroon) strains were grown as previously described [28-30] in RPMI 1640 supplemented with 0.5% Albumax II (Gibco), 25 mM sodium carbonate, 25 mM HEPES, glucose 2 g/l. Red blood cells (RBCs) were incubated in 24-well plates at 37°C in an incubator filled with a gaz phase of 5% O_2_, 5% CO_2_, 90% N_2_. Five-hundred μl of medium were used per well with 50 μl of pelleted RBCs from patients or from continuous cultures. Continuous cultures were synchronized using standard sorbitol procedure conducted twice at 48-hour intervals. For patients attending dispensaries with clinical signs of malaria, malaria attack was confirmed by PfLDH rapid test, and 5 ml periphery venous blood samples were collected after informed consent. Leukocytes were carefully removed by washing blood with medium five times and by removal of the buffy coat. Parasitaemia was determined using Giemsa-stained thin blood smears, for 50 fields at ×1,000 magnification. Field isolates were tested directly from patients, without previous cultivation or cryopreservation, in less than 48 h after sampling.

### Labelling of infected red blood cells for flow cytometry

**Figure 1:**
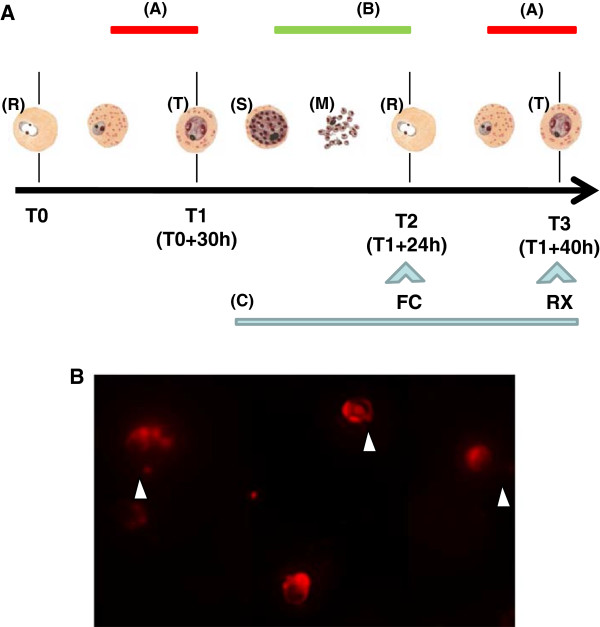
**Protocol of measurement of the effect of drugs. A)** Life cycle of the parasite: Ring stages of the parasites (R) collected in the blood of patients are cultured for 30 hours without drug to the trophozoite stage (T0). Measurement of inhibition of maturation by a drug occurs during the trophozoite stage (A). It can be detected using radio-labelling of the parasite (RX) or flow cytometry (FC). Inhibition of re-invasion (B) occurs during the schizont (S)/merozoite (M)/ring (R) transition. It can be detected after 24 hours of incubation with drug (C) by flow cytometry (FC), or coupled with inhibition of maturation, after 40 hours of incubation of drug (using either RX or FC methods). **B)** Labelling of PRCB using hydroethidine; (arrow head: parasites in the erythrocytes).

**Figure 2:**
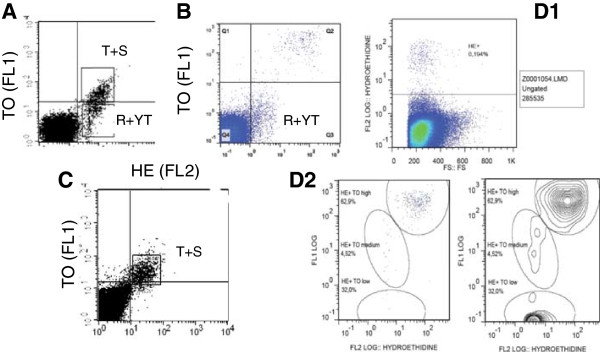
**Analysis of parasitized red blood cells using flow cytometry.** PRBC are stained with thiazole (FL1) and hydroethidine (FL2). **A-B-C/**FL1/FL2 dot plots. Upper right: quarter late stages (T + S: late trophozoites, schizonts); lower right: early stages (R + YT, rings, young trophozoites). **A/**asynchronous culture, **B/**only young stages of the parasite, **C/**same culture 30 hours later (only late stages of the parasite). **D/**Automatic analysis of the data using FlowJo software with tracking of the clouds; D1: FL2/FS analysis of hydroethidine-labelled cells (HE+). D2 FL1/FL2 plot with HE + cells gated in D1. HE + TO low: Ring; HE + TO medium: young trophozoite; HE + TO high schizonts and late trophozoites. (T + S)/(R + TY) ratio can be used to calculate inhibition of maturation or re-invasion.

The labelling of parasitized RBCs (PRBC) was performed in the dark without permeabilization of the cells in two steps [10,13,31], using two nucleic acids staining: i) vital dye hydroethidine (HE) (Interchim 17084), which is metabolized into ethidium by esterases in intact PRBC (ethidium labelling of nucleic acids results in a red fluorescence emission) [11] (Figure 1B); and, ii) thiazole orange (TO) (Sigma 17237), which binding both to RNA and DNA emitting a green fluorescence [15]. HE is prepared at 10 mg/ml in dimethyl sulphoxide then stored at −20°C. Staining is done at 37°C by adding HE to cells at a final concentration of 40 μg/ml in phosphate buffer saline (PBS) for 20 min in the dark. After two washes in PBS-SVF2% and centrifugation, PRBC were suspended in 200 μl of TO (1:20,000) for 10 min and washed again. Analysis of the samples was done using a one laser BD-Facscalibur cytometer or a Beckman Coulter. For each sample, 500,000 cells were analysed in a FL1 (TO)/FL2 (HE) dotplot for rings (R), young trophozoites (YT), trophozoites (T), and schizonts (S) (Figure 2A-B-C). Automatic analysis was also performed using FlowJo® software (Figure 2D). Uninfected RBCs were detected in the lower left corner of the cytogram (less than ten as fluorescence intenity for the two dyes).

### Comparison of parasitaemia measured by flow cytometry and Giemsa staining

To compare determination of parasitaemia using Giemsa-stained smears and flow cytometry, setting of the cytometer was done using: i) highly synchronized *in vitro* culture of *P. falciparum* 3D7; or, ii) field isolates from patients suffering from a malaria attack. To estimate parasitaemia in whole blood by flow cytometry, three plots were used: i) forward scatter *versus* HE cytogram; ii) HE histogram; and, iii) HE *versus* TO cytogram. The PRBC were gated and counted in each plot and the average of the three PRBC counts was done.

Leukocytes were excluded from the analysis by gating out highly fluorescent cells as they usually harbour greater fluorescence intensity than PRBC (gate G1 > 10^3^) [31].

### Inhibition of maturation

*In vitro* drug sensitivities measured by flow cytometry and by conventional [^3^H] hypoxanthine incorporation assay were compared on both cultured strains and field isolates. Microtests were performed as described by Desjardins and modified by Lebras *et al*. [2]. Two identical microtiter plates were prepared in parallel for both flow cytometry and radioactive analyses. The microtiter plates were coated with serial dilution of chloroquine in triplicate. Cells were incubated in 200 μl of RPMI 1640 supplemented with 0.5% Albumax II (Gibco), 25 mM sodium carbonate, 25 mM HEPES at 0.5% parasitaemia, 2.5% haematocrit, for 42 hours at 37°C in an incubator. For radio-labelling 0.5 μCi/well of [^3^H] hypoxanthine was added at the beginning of the test and cells were harvested onto glass fibre filters after 42 hours. Radioactivity was counted using a Wallac Betacount. For cytometry, culture medium was removed from the plate at the end of incubation and cells were washed and labelled as described. For each drug concentration, per cent of inhibition of the parasites was calculated according to the control wells without drug. Concentration inhibiting 50% of the parasites (IC_50_) was calculated after probit/log transformation of the dose/response curve [32].

### Inhibition of re-invasion

Using flow cytometry, inhibition of re-invasion of the red cells by the parasite can be distinguished from inhibition of maturation by incubating drug for 24 hours with late schizont infected RBCs (Figure 1A). If re-invasion occurs properly, after 24 hours of culture ring stage infected RBC must be predominant in culture. Analysis of the parasitaemia and/or of the ratio early/late stages of the parasites can quantify effect on re-invasion. Prior to incubation with drug, field isolates were cultured for 30 hours to the late stage (T1). PRBC were then cultured for 24 hours more (T2) with drug and flow cytometry analysis was done as described. To analyse the effect of the drug on the whole parasite cycle, i.e. including effect on maturation, the second plate of PRBC was incubated for 24 hours more (T3) with drug and [^3^H] hypoxanthine. Culture conditions, medium and analysis were the same as described for chloroquine. However for field isolates, due to the low amount of blood collected from children, only four concentrations (0/50/200/400 nM) of quinine have been used and percent of inhibition have been calculated directly for each one. Diisopropylfluorophosphate (DFP) was used as positive control of inhibition of re-invasion at 10 μM.

## Results

### Standardization of a flow cytometry method for the detection of parasites

**Figure 3:**
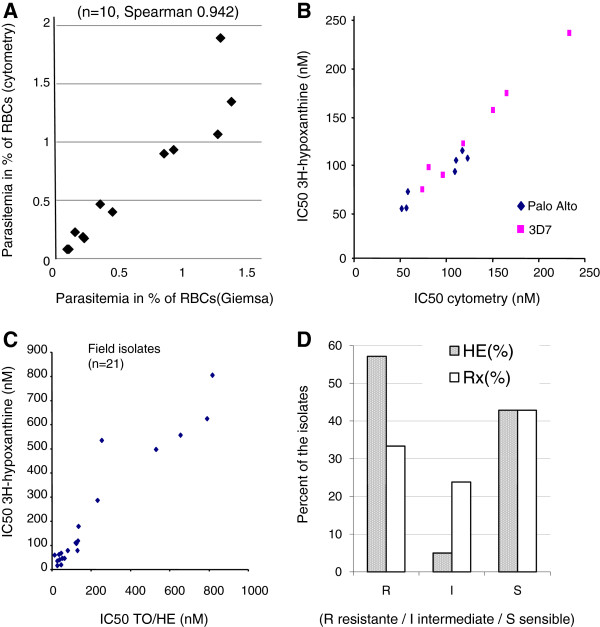
**Comparison of inhibitory concentration 50% (IC**_**50**_**) calculated in vitro using **_**3**_**H-hypoxanthine, and TO/HE methods.** Parasites were cultivated in standard microtest conditions. IC_50_ was calculated for chloroquine, using probit log transformation of the dose/effect curve. **A/**Comparison of parasitaemia calculated using microscopy and TO/HE method. Ten field isolates were analysed. Parasitaemia are expressed as per cent of RBCs. Spearman correlation test between the two methods was 0.942. **B/**Sensitivity (IC50 in nM) of Palo alto and 3D7 strains calculated for chloroquine using _3_H-hypoxanthine and TO/HE methods. Seven independent experiments were done for each strains. **C/**IC50 (in nM) of field isolates of *Plasmodium falciparum* for chloroquine, calculated using TO/HE or 3H hypoxanthine methods (21 isolates). **D/**classification of the field isolates as sensitive/intermediate/resistant strains using the two methods.

To estimate the parasitaemia in whole blood, count was done on the three gates of the HE/TO histogram and compared with the count done on the Giemsa-stained smear. In Figure 3A ten field isolates were analysed from 0.01 to 2% of parasitaemia. A Spearman correlation was found at 0.94 (p < 0.01).

### Cytometry analysis of inhibition of maturation

This study compared HE/TO labelling with standard [_3_H]-hypoxanthine test on both cultured strains and fields isolates for the determination of effect of chloroquine. As the two methods do not consider the same biological events in the parasite, concentration inhibiting 50% of the parasites were compared and not individual data obtained for each drug concentration. Seven independent experiments were done for cultured strains (Figure 3B). Mean IC_50_ ranged from 55.7 and 133.7 nM and from 93 to 230 nM for Palo Alto and 3D7 strains, respectively. Correlation between the two techniques was 96 and 99% for PA and 3D7 strains, respectively. In the same line, 26 field isolates were analysed but due to lake of maturation of the parasites, four isolates were excluded from analysis. For the 22 tests analysed (Figure 3C) the correlation between the two assays was 0.95. According to usual threshold of IC_50_ for sensitive (IC_50_ < 80 nM), intermediate (IC_50_ between 80 to 120 nM) and resistant (IC_50_ > 120 mM) isolates for chloroquine (National Reference Centre for Malaria Sensitivity, Hôpital Bichat, Paris), 54% of isolates were found resistant by the cytometry method and 41% were sensible, while 33% of isolates were resistant, 24% intermediary and 43% sensitive by isotopic assay, respectively (Figure 3D). Results were thus similar for the two assays. Discrepancy was observed for intermediate strains that were found more resistant with flow cytometry method.

### Cytometry analysis of reinvasion inhibition

**Figure 4:**
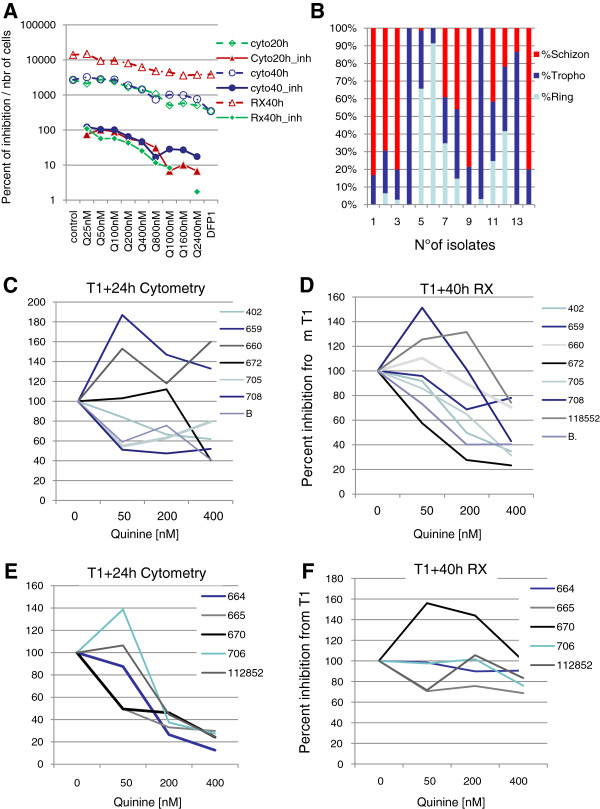
**Effect of quinine on maturation and reinvasion of parasites. A/**Sensitivity of FCM29 strain to quinine after 24 or 40 hours of incubation with the drug. Standard microtests were performed in culture. Inhibition of re-invasion/maturation was detected after 20 or 40 hours of culture, respectively. (Cyto20, count of parasites after 20,hours of incubation with drug using TO/HE method, cyto20_inh, per cent of inhibition related to T0 after 20 hours; cyto40, count of parasites after 40 hours of incubation with drug using TO/HE method; cyto40_inh per cent of inhibition related to T0 after 40 hours; RX40 count of parasites after 40 hours of incubation with drug using _3_H-hypoxanthine method; rx40_inh per cent of inhibition related to T0 after 40 hours using _3_H-hypoxanthine method). **B/**Stage specific parasitaemia measured in field isolates after 30 hours of culture. Infected RBCs from patients were cultured for 30 hours. Counts of parasites were done separately for ring, trophozoite and schizont stages, respectively. **C-D-E-F/**Inhibition of the field isolates by quinine, Per cents of inhibition related to T0 were plotted separately for each isolate using TO/HE detection method after 24 hours of incubation with drug **(C-E)** or using _3_H-hypoxanthine detection method after 40 hours of incubation with drug **(D-F)**. **C-D** isolates with maturation to schizonts in culture prior incubation with drug (i.e., **B/**) during this step quinine is not supposed to be efficient on the parasite and reinvasion can occur with an increase in parasitaemia if not inhibited by the drug; **E-F** isolates without maturation to schizonts in culture prior incubation with drug (i.e., **B/**). Quinine was used at 0/50/200/400 nM.

Effect of quinine was first assessed on FCM29 strain. When the drug is applied to schizonts over 40 hours (re-invasion plus maturation inhibition) the IC_50_ was 180 nM. When analysis was performed over 40 hours the inhibition detected was in the same range (i.e. 220 nM) (Figure 4A). However, after only 20-hours incubation with drug the inhibition detected by cytometry was similar as the 40-hour one (i.e. 205 nM).

In a second step, this assay was done with field isolates. Field isolates were first incubated in culture media for 30 hours to obtain late stage of the parasites. Seven out of the 14 isolates maturated into late stage during this time and were ready for re-invasion assay (Figure 4B). Per cents of inhibition obtained after 24 hours (using cytometry method, Figure 4C) and those obtained after 40 hours’ incubation (using Rx-method, Figure 4D) were compared. For the seven isolates with schizont at the beginning of the test, after 40 hours’ incubation, inhibition ranged from 80 to 30% at 400 nM quinine (Figure 4D). However for five out of seven, 50% of inhibition was already observed at 24 hours (Figure 4C). When isolates with early stages (parasites without clear maturation at T1, Figure 4A) were incubated in the same way with drug for 40 hours, the usual rate of inhibition by quinine was obtained (i.e., 50–200 nM, Figure 4E). No inhibition was observed at 24 hours by flow cytometry (Figure 4F).

## Discussion

This study proposes a simple protocol to study re-invasion in field isolates. For re-invasion inhibition, a couple of techniques were recently reported in the literature. However, none of them was used on field isolates but only cultured strains. Exploring other phase than maturation of the parasite is urgent to define new targets for drug [33]. Conventional [_3_H] hypoxanthine incorporation assay is easy and fast to perform, but it is limited by the price of equipment and difficulties to properly manage radio compounds, especially in developing countries. Limitation is also intrinsically linked to the biological event detected, i.e., division of DNA, which cannot be used to explore the “schizont to ring” phase of the biological life cycle of the parasite. As shown in the present study, flow cytometry using fluorescent dye targeting DNA and RNA can be used to detect separately the different stages of the parasite, and analyse the parasite’s re-invasion phase. To analyse the inhibition of maturation by drugs, correlation between conventional [^3^H] hypoxanthine incorporation assay and the latter method as well as the reproducibility of the proposed approach, seven independent experiments were performed using *P. falciparum* Palo Alto (PA) strain and 3D7 clone in the presence of chloroquine. A very high agreement between findings of the two techniques was observed, strongly suggesting that the proposed protocol is suitable to study re-invasion in field isolates

However, discrepancy was observed for intermediate strains, which were found more resistant with flow cytometry method. This can be due to the labelling of both RNA and DNA by fluorescent dye despite labelling of only DNA by 3H-hypoxanthine.

Quinine was found efficient to modulate schizont-ring transition on 3D7 strain, but only on five out of the seven field isolates used. Other puzzling observations have been previously reported, as for Wilson *et al*. [31] who found no effect of quinine in their re-invasion experiments. Considering mechanistic studies revealing quinine’s ability to target erythrocyte channels [27], this difference can be due to the polymorphism of red cells used in the different experiment and especially to the sensitivity of channels to drugs. The effect of quinine on the transition schizont-ring (including re-invasion step) supported by the findings of the present study are in line with early work of Hommel *et al*. [28]. Noticeably, when drug was applied to schizonts over 40 hours (re-invasion plus maturation inhibition) IC_50_ was in the same range in both [_3_H] hypoxanthine incorporation assay and flow cytometric method using TO and HE. However, such results were already observed only 20 hours after incubation with drug, suggesting a great part of release/re-invasion inhibition in the final result.

Flow cytometric method using TO and HE for the detection of parasites in whole blood is simple, fast and cheap, and can be used with field isolates. For both cultured strains and field samples, the cytometry test harboured a good reproducibility and a good correlation with the radioactive one. It can also be used to analyse both inhibition of maturation and of re-invasion of the red cells.

## Competing interests

The authors declare that they have no competing interests.

## Authors’ contributions

MLV and RR developed the method and tested drug sensitivity of the isolates. JCB and DA participated in its design and in the statistical analysis. RJ conceived of the study, participated in the design of the study and performed the statistical analysis. All authors read and approved the final manuscript.
